# Is avoidable diabetes-related hospitalization in older patients with type 2 diabetes mellitus associated with increased health expenditure?: A nationwide retrospective cohort study in South Korea

**DOI:** 10.1016/j.pmedr.2024.102946

**Published:** 2024-12-15

**Authors:** Woo-Ri Lee, Gyeong-Min Lee, Noorhee Son, Kyu-Tae Han, Sungyoun Chun, Yehrhee Son, Ki-Bong Yoo

**Affiliations:** a Department of Research and Analysis, National Health Insurance Service Ilsan Hospital, Goyang, South Korea; b Department of Premedical, College of Medicine, Dankook University, Cheonan, South Korea; c Division of Cancer Control and Policy, National Cancer Control Institute, National Cancer Center, Goyang, South Korea; d ESON Medical Management Institute, ESON Hospital, Ulsan, South Korea; e Division of Health Administration, College of Software and Digital Healthcare Convergence, Yonsei University, Wonju, South Korea

**Keywords:** Type 2 diabetes mellitus, Avoidable diabetes-related hospitalization, Health expenditure, Older patients, IPTW

## Abstract

**Objective:**

With South Korea's population aging rapidly, the number of patients with type 2 diabetes mellitus (T2DM) is expected to rise, leading to worsened health outcomes and potentially straining healthcare financing. This study aimed to investigate how avoidable diabetes-related hospitalizations affect short- and long-term health expenditures.

**Methods:**

Data from the National Health Insurance Service-Senior cohort from 2008 to 2019 in South Korea. A total of 27,081 participants aged 60 years and older who were diagnosed with T2DM were included in the study. The independent variable in this study was avoidable diabetes-related hospitalization according to the ICD-10 criteria “E11”. The outcome measures included one- and five-year health expenditures. Regression analysis was performed using the generalized estimating equation (GEE) with a gamma distribution and log-link function. Inverse Probability of Treatment Weighting (IPTW) analysis was conducted to enhance the robustness of the results.

**Results:**

Out of the 27,081 participants, 685 patients (2.5 %) experienced avoidable diabetes-related hospitalizations. GEE analysis with IPTW weights revealed that participants who experienced avoidable hospitalizations had a higher risk of increased health expenditures (one-year: relative risk (RR) 1.83, 95 % CI 1.76–1.91; five-year: RR 1.63, 95 % CI 1.57–1.69). Consistent patterns were observed even without weighting (one-year: RR 1.85, 95 % CI 1.68–2.04; five-year: RR 1.60, 95 % CI 1.47–1.74).

**Conclusions:**

Our findings highlight the importance of continuous health management to prevent avoidable hospitalization, thereby promoting health and ensuring the financial stability of older patients with T2DM within the healthcare insurance system.

## Highlights

• Avoidable diabetes-related hospitalizations increase health costs in older patients with type 2 diabetes mellitus.
• Continuous diabetes management reduces hospitalization risk.
• Improving care continuity may lower hospitalization rates and associated expenses.

## 1 Introduction

With South Korea's population aging rapidly, the prevalence of type 2 diabetes mellitus (T2DM) is increasing (Cannon et al., 2018; Khan et al., 2020). Globally, approximately 462 million patients were suffering from T2DM in 2017 (Khan et al., 2020). In Korea, approximately 30 % of adults aged 65 years and older had diabetes (Bae et al., 2022). Diabetes is considered one of the top ten causes of death globally and accounts for over 80 % of all premature non-communicable disease deaths, posing significant health burdens (Lin et al., 2020). Older patients with diabetes, in particular, belong to a high-risk group that requires extra attention (Mohammad et al., 2021). Diabetes imposes health burdens on patients and socio-economic burdens. In 2019, global direct health expenditure on diabetes was estimated to be approximately USD 760 billion (Williams et al., 2020). According to previous studies, diabetes-related direct and indirect costs in Korea were estimated at USD 1.2 billion, indicating a significant burden of diabetes in Korea (Oh et al., 2021). The economic burden of diabetes has significantly increased over recent decades and is expected to rise in the future (Lin et al., 2020). The increasing burdens related to diabetes, along with the rise in the number of patients with T2DM, have become major concerns in global healthcare (Khan et al., 2020).

T2DM is a typical ambulatory care-sensitive condition, which includes conditions in which timely primary healthcare can prevent hospitalization and complications (Jung and Lee, 2023). Therefore, hospitalization owing to T2DM can be referred to as avoidable hospitalization. However, in Korea, the number of cases with sex-age standardized avoidable diabetes-related hospitalizations per 100,000 population was 340, which was approximately three times higher than the Organisation for Economic Co-operation and Development (OECD) average of 114 cases (Organisation for Economic Co-operation and Deveopment, 2023b). Previous studies have shown that avoidable hospitalizations contribute to increased health expenditures in the general population (Rocha et al., 2020). Moreover, problems such as deterioration in health caused by the occurrence of complications can lead to increased health expenditures associated with T2DM (Wu et al., 2012). Despite the persistent increase in health expenditure and high rates of avoidable hospitalizations owing to T2DM, there is a lack of studies investigating the impact of avoidable hospitalizations on health expenditure. Therefore, our study aimed to investigate the short- and long-term associations between avoidable diabetes-related hospitalization and health expenditure.

## 2 Methods

### 2.1 Data and study population

In South Korea, efforts to achieve universal health coverage have led to a continuous strengthening of health insurance benefits. Accordingly, in 1989, the National Health Insurance Service (NHIS) successfully achieved nationwide coverage of medical services for all citizens (Kwon, 2009; NHIS, 2024b). The country operates a NHI system, with the NHIS serving as a single insurer. All citizens, excluding those receiving medical aid, are required to contribute insurance premiums pooled to distribute risk equitably (Kwon, 2009). Health expenditures are the sum of out-of-pocket payments and insurer's contribution. If healthcare services are needed, citizens receive healthcare at hospitals, with a portion of the costs paid out-of-pocket. The NHIS covers the remaining expenses to ensure financial protection for the insured (Kwon, 2009). Therefore, NHIS data is representative and allows for the examination of patients' histories, utilization of medical services, and more for the entire population (Kim et al., 2019). In this study, we analyzed NHIS-Senior cohort data provided by the NHIS from 2008 to 2019. In the case of NHIS-Senior cohort data, the population aged 60 years or older as of 2008 was stratified based on sex, age, healthcare insurance premiums, and regional area, and 8 % were randomly sampled (Kim et al., 2019).

**Fig. 1:**
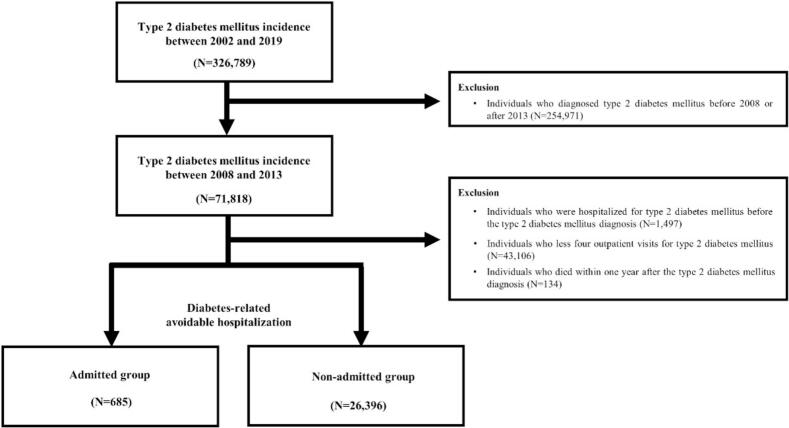
Study participants' selection process: National Health Insurance Service–Senior Cohort, 2008–2019, South Korea.

In this study, participants primarily diagnosed with T2DM using the International Classification of Diseases, Tenth Revision (ICD-10) criteria “E11” were included (*N* = 326,789). Among these participants, those diagnosed with T2DM before 2008 or after 2013 (*N* = 254,971), those hospitalized for T2DM before the initial diagnosis of T2DM (*N* = 1497), those with fewer than four outpatient visits within one-year of T2DM diagnosis (*N* = 43,106), and those who died within one-year after T2DM diagnosis (*N* = 134) were excluded. Finally, 27, 081 participants were included in this study (Fig. 1).

### 2.2 Variables

#### 2.2.1 Outcome measures

The outcome measure in this study was health expenditure. To explore the long- and short-term effects of avoidable diabetes-related hospitalization, health expenditures were measured for one- and five-year following the observation of avoidable diabetes-related hospitalization. To account for annual inflation, we applied the annual conversion index inflation rate used in the fee-for-service system to calculate health expenditure (National Health Insurance Service, 2024a). The annual inflation rate reflects the year 2019, the last observable point in this study. The currency was converted to USD using the World Bank's 2019 currency data to provide a broader interpretation of health expenditure (World Bank Group, 2024). After aggregating health expenditures for both the long and short periods, participants were divided into observation periods. Subsequently, each period was calculated by multiplying 365 by 1825 days. The formula for calculating health expenditure used in this study is as follows:

Equation 1 . One-year health expenditure = (sum of health expenditure [cost × annual conversion index inflation rate]) ÷ (sum of the observation period [365 days from one-year after T2DM diagnosis]) × 365.

Equation 2 . Five-year health expenditure = (sum of health expenditure [cost × annual conversion index inflation rate]) ÷ (sum of the observation period [1825 days from one-year after T2DM diagnosis]) × 1825.

#### 2.2.2 Independent variable

**Fig. 2:**
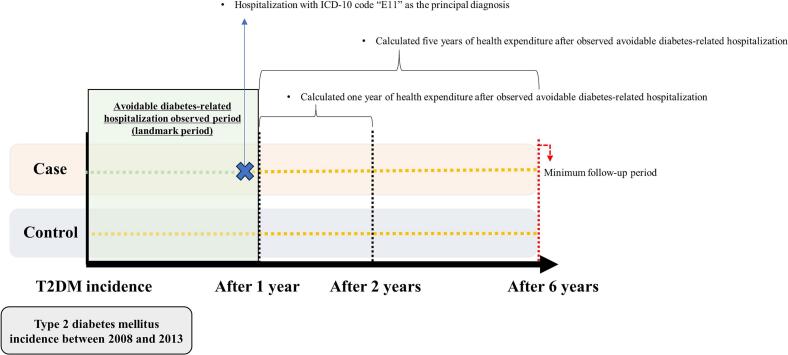
Study timeline: National Health Insurance Services–Senior Cohort, 2008–2019, South Korea.

The independent variable in this study was avoidable diabetes-related hospitalization. According to the Organisation for Economic Co-operation and Development (OECD), avoidable hospitalizations for T2DM are defined as admissions with a principal diagnosis coded as E11.x under the ICD-10 criteria for patients aged 15 years or older (OECD, 2023a). Based on this definition, in this study, we considered avoidable diabetes-related hospitalization as any admission with a principal diagnosis of E11 occurring within one year of T2DM diagnosis (Fig. 2).

#### 2.2.3 Covariates

In this study, covariates were categorized into socioeconomic, health-related, and diabetes-related factors. Socioeconomic factors included sex (male or female); age (60s, 70s, or ≥ 80s); income (≤20 %, 21–50 %, 51–80 %, 81–100 %), region (Seoul, Gyeonggi, Metropolitan, or other cities), and type of healthcare insurance (medical aid or NHI). In this study, income was represented using the NHI premium, which is calculated based on an individual's income (NHIS, 2024c). For the NHIS-Senior cohort data, insurance premiums are categorized into 11 groups, including Medical Aid beneficiaries and 10 additional categories segmented by 10 % increments (NHIS, 2024c). This variable was restructured into four groups for the purpose of this study. In South Korea, medical resources are regionally concentrated in metropolitan and large urban areas, reflecting significant regional disparities in infrastructure and resource availability (Eun, 2022; Statistics Korea, 2023). The regions were categorized into four groups to capture these characteristics: (1) Seoul, the capital city and central hub for medical and administrative services; (2) Gyeonggi, the surrounding area within Seoul, characterized by high connectivity and shared infrastructure with Seoul; (3) Metropolitan areas, which include six large cities (Busan, Daegu, Incheon, Gwangju, Daejeon, and Ulsan) designated as metropolitan areas; and (4) Other cities, encompassing the remaining areas. Health-related factors included disability (non-disabled, disabled) and Charlson Comorbidity Index (CCI) scores (0, 1, 2, and ≥ 3). The diabetes-related factors considered in this study included the Diabetes Complication Severity Index (DCSI), continuity of care (COC), and year of T2DM diagnosis. DCSI serves as an indicator of diabetes-related complications, assigning weights to seven diseases: Acute metabolic complication, cardiovascular disease, cerebrovascular disease, nephropathy, neuropathy, peripheral vascular disease, and retinopathy (Yoo et al., 2020). Scores were categorized into four groups (0, 1, 2, and ≥ 3). COC is an index indicating the continuous management of chronic diseases and ranges between 0 and 1; the closer it is to 1, the better the continuous management (Jung and Lee, 2023). COC variables were classified into those with a COC score of 1 (good) and less than 1 (bad). The year of the T2DM diagnosis was the participant's initial T2DM diagnosis year. Other factors included all-cause death, with the presence or absence of death considered only if it occurred within the health expenditure calculation period.

### 2.3 Statistical analysis

Avoidable diabetes-related hospitalization, which was the independent variable in this study, is a time-dependent variable that may occur repeatedly over time. To effectively control for the influence of time-dependent variables (Morgan, 2019), we opted for analysis using the landmark method. In the landmark method, only the presence or absence of exposure during a specific observation period is considered, without accounting for the possibility of exposure after the specified period (Morgan, 2019). In this study, the exposure observation period was set to one-year because of the application of the landmark method.

The analysis was conducted in four stages. First, to assess differences in health expenditures based on covariates, we performed *t*-tests and analysis of variance (ANOVA) analyses. The statistical significance of the t-test and ANOVA was verified using a threshold of *p* < 0.05. Second, we conducted a regression analysis using a Generalized Estimating Equation (GEE) model. The GEE model was applied with a gamma distribution and log link to minimize bias in the analysis results (Kuss, 2015). The GEE analysis was defined as statistically significant when the lower limit of the 95 % confidence interval (CI) for the measure exceeded one. Third, we employ the Inverse Probability of Treatment Weighting (IPTW) method to enhance the level of evidence for the analysis results. The homogeneity after applying the IPTW of the study participants was assessed, and verification was based on an absolute value of the standardized difference of 0.1 (Faries et al., 2020). Fourth, we performed a sensitivity analysis to improve the robustness of the results. Sensitivity analysis was conducted in the following two main ways: a) changing the observation period of the independent variable to two- and three-year and b) altering the definition of patients with T2DM to those with two or more outpatient visits within one-year of T2DM diagnosis. All regression analyses were conducted while adjusting for all covariates. All the analyses were performed for 2023 using SAS version 9.4 (SAS Institute, Cary, NC, USA).

## 3 Results

Out of the total 31,222 study participants, 758 (2.4 %) experienced avoidable diabetes-related hospitalizations (Table 1). The average health expenditure based on the participants' general characteristics is presented in Table 2. The one-year average health expenditure of the study participants was 2194 USD, while the five-year average health expenditure was 12,483 USD. For the one-year average health expenditure, participants who experienced avoidable diabetes-related hospitalization incurred 4674 USD, whereas those who did not experience incurred 2130 USD of expenses; the one-year average health expenditure for those with avoidable diabetes-related hospitalization history was significantly larger (*p* < 0.01). For the five-year average health expenditure, participants who experienced avoidable diabetes-related hospitalization incurred 23,614 USD, whereas those who did not experience incurred 12,194 USD of expenses; the five-year average health expenditure for those with avoidable diabetes-related hospitalization history was significantly larger (p < 0.01).

**Table 1:** General characteristics of the study population by avoidable diabetes-related hospitalization using the National Health Insurance Services–Senior cohort 2008–2019 in South Korea.

**Variable**⁎	**Total**	**Avoidable diabetes-related hospitalization**				
		**Yes**		**No**		***p*****-value**⁎⁎
		**N**	**%**	**No**	**%**	
**Total**	27,081	685	(2.5)	26,396	(97.5)	
**Sex**						
Male	12,212	291	(2.4)	11,921	(97.6)	0.16
Female	14,869	394	(2.6)	14,475	(97.4)	
**Age**						
60s	15,703	345	(2.2)	15,358	(97.8)	<0.01
70s	10,063	282	(2.8)	9781	(97.2)	
≥80s	1315	58	(4.4)	1257	(95.6)	
**Income**						
≤20 % (lowest)	6590	230	(3.5)	6360	(96.5)	<0.01
21 %–50 %	4750	108	(2.3)	4642	(97.7)	
51 %–80 %	7707	147	(1.9)	7560	(98.1)	
81 %–100 % (highest)	8034	200	(2.5)	7834	(97.5)	
**Region**						
Seoul	5356	80	(1.5)	5276	(98.5)	<0.01
Gyeonggi	5227	114	(2.2)	5113	(97.8)	
Metropolitan	6660	169	(2.5)	6491	(97.5)	
Other cities	9838	322	(3.3)	9516	(96.7)	
**Type of healthcare insurance**						
Medical Aid	2172	113	(5.2)	2059	(94.8)	<0.01
National Health Insurance	24,909	572	(2.3)	24,337	(97.7)	
**Disability**						
Non-disabled	23,018	558	(2.4)	22,460	(97.6)	<0.01
Disabled	4063	127	(3.1)	3936	(96.9)	
**CCI**						
0	9590	165	(1.7)	9425	(98.3)	<0.01
1	4727	107	(2.3)	4620	(97.7)	
2	6465	157	(2.4)	6308	(97.6)	
≥3	6299	256	(4.1)	6043	(95.9)	
**DCSI**						
0	24,069	558	(2.3)	23,511	(97.7)	<0.01
1	1766	58	(3.3)	1708	(96.7)	
2	751	26	(3.5)	725	(96.5)	
≥3	495	43	(8.7)	452	(91.3)	
**Continuity of Care**						
Bad (<1)	8007	396	(4.9)	7611	(95.1)	<0.01
Good (=1)	19,074	289	(1.5)	18,785	(98.5)	
**Death within one-year**						
No	26,610	655	(2.5)	25,955	(97.5)	<0.01
Yes	471	30	(6.4)	441	(93.6)	
**Death within five-year**						
No	24,457	534	(2.2)	23,923	(97.8)	<0.01
Yes	2624	151	(5.8)	2473	(94.2)	
**Diagnosed year of T2DM**						
2008	4435	124	(2.8)	4311	(97.2)	0.10
2009	4329	120	(2.8)	4209	(97.2)	
2010	4351	126	(2.9)	4225	(97.1)	
2011	4762	113	(2.4)	4649	(97.6)	
2012	4770	105	(2.2)	4665	(97.8)	
2013	4434	97	(2.2)	4337	(97.8)	

⁎ *CCI: Charlson comorbidity index; DCSI: Diabetes complication severity index; T2DM: Type 2 diabetes mellitus.*

⁎⁎ *p-value were generated using the chi-squared test.*

**Table 2:** Health expenditure after observed avoidable diabetes-related hospitalization using the National Health Insurance Services-–Senior cohort 2008–2019 in South Korea.

**Variable**⁎	**Health expenditure (Unit: USD)**					
	**One-year**			**Five-year**		
	**Mean**	**±Std**	**p-value**	**Mean**	**±Std**	***p*-value**⁎⁎
**Total**	2194	±4897		12,483	±19,157	
**Avoidable diabetes-related hospitalization**						
No	2130	±4751	<0.01	12,194	±18,633	<0.01
Yes	4674	±8495		23,614	±31,702	
**Sex**						
Male	2209	±5346	0.66	12,523	±19,973	0.76
Female	2182	±4495		12,451	±18,460	
**Age**						
60s	1925	±4506	<0.01	10,781	±17,330	<0.01
70s	2522	±5284		14,545	±20,637	
≥80s	2888	±5970		17,028	±25,001	
**Income**						
≤20 % (lowest)	2436	±4861	<0.01	13,854	±19,284	<0.01
21 %–50 %	1899	±4087		11,652	±18,185	
51 %–80 %	2107	±4802		11,770	±18,228	
81 %–100 % (highest)	2253	±5418		12,534	±20,383	
**Region**						
Seoul	1941	±4771	<0.01	11,397	±19,115	<0.01
Gyeonggi	2151	±5710		11,411	±19,443	
Metropolitan	2242	±4975		12,832	±19,531	
Other cities	2322	±4416		13,408	±18,711	
**Type of healthcare insurance**						
Medical Aid	3158	±5332	<0.01	17,426	±21,338	<0.01
National Health Insurance	2110	±4849		12,052	±18,894	
**Disability**						
Non-disabled	2027	±4380	<0.01	11,757	±17,918	<0.01
Disabled	3137	±7080		16,596	±24,648	
**CCI**						
0	1642	±4007	<0.01	9890	±16,057	<0.01
1	2192	±4183		12,719	±16,766	
2	2027	±4551		12,009	±19,110	
≥3	3207	±6540		16,740	±23,896	
**DCSI**						
0	2224	±4875	<0.01	12,715	±19,353	<0.01
1	1807	±4972		9942	±16,476	
2	1784	±3975		10,132	±15,441	
≥3	2750	±6568		13,834	±22,385	
**Continuity of Care**						
Bad (<1)	2451	±5046	<0.01	13,656	±19,686	<0.01
Good (=1)	2086	±4830		11,991	±18,909	
**Death**						
No	2170	±4836	<0.01	11,464	±17,484	<0.01
Yes	3527	±7495		25,011	±30,842	
**Year of T2DM Diagnosis**						
2008	2091	±4474	0.38	12,189	±19,435	<0.01
2009	2251	±4941		11,765	±17,461	
2010	2185	±4871		12,165	±18,204	
2011	2302	±5403		12,839	±19,772	
2012	2180	±4900		12,650	±19,123	
2013	2149	±4707		13,229	±20,652	

⁎ *CCI: Charlson comorbidity index; DCSI: Diabetes complication severity index; T2DM: Type 2 diabetes mellitus.*

⁎⁎ *p-value were generated using the t-test or analysis of variance.*

The GEE analysis results aimed at confirming the association between avoidable diabetes-related hospitalization and health expenditures are presented in Table 3. When applying IPTW weights before proceeding with the GEE analysis, validation was performed based on a standardized difference absolute value of 0.1 to confirm the homogeneity of the case-control population (Supplementary Table 1). In the unweighted model, both one- and five-year health expenditures were higher for participants who experienced avoidable diabetes-related hospitalization compared to those who did not (one-year: relative risk (RR) 1.85, 95 % CI 1.68–2.04; five-year: RR 1.60, 95 % CI 1.47–1.74). In the model that applied IPTW weights, participants with avoidable diabetes-related hospitalization experienced higher health expenditures at one- and five-year compared to those who did not (one-year: RR 1.83, 95 % CI 1.76–1.91; five-year: RR 1.63, 95 % CI 1.57–1.69). The results confirming the trend using a normal distribution are shown in Supplementary Table 2.

**Table 3:** Generalized estimating equations analysis on the association between avoidable diabetes-related hospitalization and health expenditure using the National Health Insurance Services-–Senior cohort 2008–2019 in South Korea.

**Variable**⁎	**Health expenditure**							
	**Unweighted model**				**IPTW weighted model**			
	**One-year**		**Five-year**		**One-year**		**Five-year**	
	**RR**	**95 % CI**	**RR**	**95 % CI**	**RR**	**95 % CI**	**RR**	**95 % CI**
**Avoidable diabetes-related hospitalization**								
No	1.00		1.00		1.00		1.00	
Yes	1.85	(1.68–2.04)	1.60	(1.47–1.74)	1.83	(1.76–1.91)	1.63	(1.57–1.69)
**Sex**								
Male	1.00		1.00		1.00		1.00	
Female	0.97	(0.94–1.01)	1.01	(0.98–1.03)	0.96	(0.93–0.99)	1.00	(0.97–1.02)
**Age**								
60s	1.00		1.00		1.00		1.00	
70s	1.23	(1.19–1.27)	1.24	(1.20–1.27)	1.26	(1.22–1.31)	1.23	(1.19–1.26)
≥80s	1.37	(1.27–1.47)	1.29	(1.22–1.38)	1.41	(1.32–1.52)	1.39	(1.31–1.48)
**Income**								
≤20 % (lowest)	1.00		1.00		1.00		1.00	
21 %–50 %	0.90	(0.85–0.95)	0.99	(0.94–1.03)	0.86	(0.81–0.91)	0.97	(0.93–1.02)
51 %–80 %	1.01	(0.96–1.06)	0.99	(0.95–1.03)	0.97	(0.93–1.02)	0.99	(0.95–1.03)
81 %–100 % (highest)	1.02	(0.97–1.07)	1.00	(0.96–1.04)	0.96	(0.92–1.01)	0.97	(0.93–1.01)
**Region**								
Seoul	1.00		1.00		1.00		1.00	
Gyeonggi	1.07	(1.02–1.12)	0.95	(0.92–0.99)	1.11	(1.05–1.17)	0.94	(0.90–0.98)
Metropolitan	1.11	(1.06–1.16)	1.07	(1.03–1.11)	1.15	(1.09–1.21)	1.07	(1.03–1.12)
Other cities	1.12	(1.07–1.17)	1.10	(1.06–1.14)	1.12	(1.07–1.17)	1.09	(1.05–1.13)
**Type of healthcare insurance**								
Medical Aid	1.00		1.00		1.00		1.00	
National Health Insurance	0.78	(0.73–0.84)	0.79	(0.75–0.84)	0.82	(0.77–0.87)	0.79	(0.75–0.84)
**Disability**								
Non-disabled	1.00		1.00		1.00		1.00	
Disabled	1.44	(1.37–1.50)	1.28	(1.24–1.33)	1.43	(1.37–1.49)	1.26	(1.22–1.31)
**CCI**								
0	1.00		1.00		1.00		1.00	
1	1.26	(1.20–1.32)	1.22	(1.17–1.26)	1.27	(1.21–1.33)	1.23	(1.18–1.28)
2	1.20	(1.15–1.25)	1.18	(1.14–1.22)	1.18	(1.14–1.24)	1.18	(1.14–1.22)
≥3	1.77	(1.70–1.84)	1.48	(1.43–1.53)	1.78	(1.71–1.86)	1.49	(1.44–1.55)
**DCSI**								
0	1.00		1.00		1.00		1.00	
1	0.85	(0.80–0.91)	0.83	(0.79–0.87)	0.92	(0.87–0.98)	0.84	(0.79–0.88)
2	0.85	(0.77–0.93)	0.84	(0.78–0.91)	0.83	(0.75–0.91)	0.84	(0.77–0.91)
≥3	1.14	(1.02–1.28)	0.98	(0.89–1.08)	1.11	(1.01–1.23)	0.94	(0.86–1.02)
**Continuity of Care**								
Bad (<1)	1.00		1.00		1.00		1.00	
Good (=1)	0.89	(0.86–0.92)	0.91	(0.88–0.93)	0.92	(0.88–0.95)	0.94	(0.91–0.96)
**Death**								
No	1.00		1.00		1.00		1.00	
Yes	1.36	(1.21–1.53)	2.25	(2.15–2.35)	1.11	(1.01–1.24)	2.02	(1.94–2.11)
**Diagnosed year of T2DM**								
2008	1.00		1.00		1.00		1.00	
2009	1.08	(1.03–1.14)	0.97	(0.93–1.02)	1.07	(1.02–1.13)	0.93	(0.89–0.98)
2010	1.04	(0.98–1.10)	0.97	(0.93–1.02)	1.05	(0.99–1.11)	0.95	(0.91–0.99)
2011	1.08	(1.03–1.14)	1.04	(0.99–1.08)	1.09	(1.03–1.15)	1.01	(0.96–1.05)
2012	1.03	(0.98–1.09)	1.03	(0.98–1.08)	1.01	(0.96–1.07)	1.00	(0.95–1.05)
2013	0.99	(1.09–1.04)	1.07	(1.03–1.12)	1.06	(1.01–1.12)	1.11	(1.06–1.17)

⁎ *IPTW: Inverse probability of treatment weighting; RR: Relative risk; CI: confidence interval; CCI: Charlson comorbidity index; DCSI: Diabetes complication severity index; T2DM: Type 2 diabetes mellitus.*

The results of the sensitivity analysis conducted to enhance the robustness of the study results are presented in Table 4. In the unweighted model, when the observation period for avoidable diabetes-related hospitalization was extended to two years, participants with avoidable hospitalizations had significantly higher health expenditure at both one (RR 2.36, 95 % CI 2.06–2.70) and five (RR 1.83, 95 % CI 1.60–2.08) years. When IPTW was applied, one- and five-year health expenditures were also greater for participants with avoidable diabetes-related hospitalization (one-year: RR 2.32, 95 % CI 2.20–2.44; five-year: RR 1.83, 95 % CI 1.74–1.92). In the unweighted model, when the observation period for avoidable diabetes-related hospitalization was extended to three years, participants with avoidable hospitalizations also showed greater health expenditure at both one (RR 2.00, 95 % CI 1.79–2.24) and five (RR 1.76, 95 % CI 1.57–1.97) years. When IPTW was applied, one- and five-year health expenditures were greater for participants who experienced avoidable diabetes-related hospitalization (one-year: RR 1.94, 95 %CI 1.85–2.03; five-year: RR 1.76, 95 % CI 1.68–1.84). After modifying the definition of T2DM to two or more outpatient visits within one-year after T2DM was diagnosed, in a model that did not apply weights, the one- and five-year health expenditures for participants who experienced avoidable diabetes-related hospitalization were greater in both one- and five-year (One-year: RR 1.87, 95 % CI 1.70–2.04; Five-year: RR 1.63, 95 % CI 1.51–1.76). When IPTW was applied, one- and five-year health expenditures were greater for participants who experienced avoidable diabetes-related hospitalization (one-year: RR 1.85, 95 % CI 1.79–1.92; five-year: RR 1.65, 95 % CI 1.60–1.71).

**Table 4:** Sensitivity analysis using the National Health Insurance Services–Senior cohort 2008–2019 in South Korea.

**Subgroup**		**Health expenditure**⁎							
		**Unweighted model**				**IPTW weighted model**			
		**One-year**		**Five-year**		**One-year**		**Five-year**	
		**RR**	**95 % CI**	**RR**	**95 % CI**	**RR**	**95 % CI**	**RR**	**95 % CI**
**Observation period for avoidable diabetes-related hospitalization**									
**Extend to two-year**	**Avoidable diabetes-related hospitalization**								
	No	1.00		1.00		1.00		1.00	
	Yes	2.36	(2.06–2.70)	1.83	(1.60–2.08)	2.32	(2.20–2.44)	1.83	(1.74–1.92)

**Extend to three-year**	**Avoidable diabetes-related hospitalization**								
	No	1.00		1.00		1.00		1.00	
	Yes	2.00	(1.79–2.24)	1.76	(1.57–1.97)	1.94	(1.85–2.03)	1.76	(1.68–1.84)

**T2DM definition (Outpatient visit over two times)**									
	**Avoidable diabetes-related hospitalization**								
	No	1.00		1.00		1.00		1.00	
	Yes	1.87	(1.70–2.04)	1.63	(1.51–1.76)	1.85	(1.79–1.92)	1.65	(1.60–1.71)

⁎ *IPTW: Inverse probability of treatment weighting; RR: Relative risk; CI: confidence interval.*

## 4 Discussion

### 4.1 Key findings

This study aimed to examine the short- and long-term associations between avoidable diabetes-related hospitalizations and health expenditure in older patients with T2DM. The analysis revealed that individuals who experienced avoidable diabetes-related hospitalizations had increased short- and long-term health expenditures. This association was consistent across the regression analyses, even after applying the IPTW and conducting sensitivity analyses.

### 4.2 Interpretation

The findings of the study align with the results of a previous study that showed increased health expenditure among those who experienced avoidable hospitalizations (Rocha et al., 2020). According to the previous literature, individuals aged 65 years and older with T2DM exhibited higher hospitalization rates, and health expenditures related to avoidable diabetes-related hospitalizations accounted for approximately 76.4 % of total diabetes-related health expenditures (Oh et al., 2021). Preventing avoidable hospitalization requires early intervention and quality healthcare services. Previous studies have indicated that better continuity of care for individuals with diabetes reduces the risk of hospitalization and is associated with a lower hospitalization rate (Christakis et al., 2001; Knight et al., 2009). Additionally, in our study, 29.6 % of patients showed poor continuity of care, with one in three patients failing to maintain consistent healthcare management (*p* < 0.01; See Table 1). Given that older patients with T2DM are more likely to experience poor continuity of care, they face an increased risk of disease progression and complications, which eventually lead to higher health expenditures (David et al., 2016; Ki et al., 2014; National Health Insurance Service, 2024a). Therefore, it is important to develop interventions aimed at effectively managing diabetes in older patients. Taiwan provides an example of comprehensive community-based initiatives known as Elderly Social Services (ESS) (Li et al., 2021). These programs facilitate social support by creating social networks for older adults and sharing beneficial knowledge. Previous studies have shown that participation in ESS can positively impact social support among older adults (Spencer-Bonilla et al., 2017) and the management of HbA1c levels among patients with T2DM within three months (Vassilev et al., 2014). Considering the substantial number of older adults with T2DM, similar community-based efforts like those in Taiwan are necessary.

The analysis revealed that individuals who experienced avoidable diabetes-related hospitalizations incurred higher short- and long-term health expenditures. Furthermore, the sensitivity analysis consistently showed similar results when the observation period for avoidable diabetes-related hospitalization was extended to two- or three-year. Consequently, effective management of chronic conditions is essential to reducing long-term health expenditures. Avoidable diabetes-related hospitalizations can arise owing to complications or metabolic disorders, leading to deteriorating health (American Diabetes Association, 2004; Jung and Lee, 2023; Vera et al., 2020). Hence, early and continuous long-term health management should be conducted to prevent avoidable hospitalizations. Previous studies have demonstrated that increased continuity of care among patients with T2DM is associated with a reduced incidence of complications, avoidable diabetes-related hospitalization, and health expenditure (Nam et al., 2019). Given that patients with T2DM are at a higher risk of experiencing diabetes-related complications as the prevalence increases, long-term, consistent healthcare management is needed (Toni et al., 2023).

T2DM accounts for approximately 90 % of all diabetes cases worldwide and represents a significant portion, accounting for approximately 86.9 % of the economic burden associated with diabetes (Oh et al., 2021). In the case of South Korea, T2DM constitutes a substantial proportion, approximately 10.6 % of the total direct medical expenses (Kim and Kim, 2022). Moreover, previous studies indicate a notable increase in direct costs associated with hospitalizations for diabetes treatment (Jinli et al., 2023; Joel, 2023; Parker et al., 2024; Soares-Andrade et al., 2023).

South Korea's health insurance policy operates on the principle of risk pooling, incorporating both horizontal and vertical redistributions. Horizontal redistribution occurs because high-income groups help alleviate the health expenditure burden on low-income groups. Similarly, vertical redistribution takes place as younger generations contribute to offsetting the health expenditure burden on older generations (Kim, 2023). However, the rapidly aging population and challenges such as declining birth rates pose threats to horizontal and vertical redistribution, affecting the stability and sustainability of South Korea's health insurance financing (Park et al., 2023). Therefore, it is important for older patients to engage in continuous management of T2DM to mitigate the likelihood of increased health expenditure, ensuring both personal health and the stability of future generations' healthcare insurance finances.

### 4.3 Limitations

This study had a few limitations. First, for patients with diabetes, it was necessary to adjust for lifestyle factors such as smoking and alcohol consumption and laboratory results such as HbA1c and fasting blood sugar. However, owing to constraints in the claim data, these variables could not be included. To overcome this limitation, we included disability and CCI scores as health-related factors and DCSI and COC variables as diabetes-related factors in the analysis. Second, there is a possibility of inconsistencies between the actual hospital diagnosis codes and those used for insurance claims. However, since patients with T2DM were defined based on at least four outpatient visits using the principal diagnosis criterion, the likelihood of errors owing to diagnosis code inconsistency was considered low. Third, avoidable diabetes-related hospitalization is a time-dependent variable that can occur even one-year after T2DM diagnosis. To overcome this limitation, we used the landmark method to effectively control for the impact of time-dependent variables.

Additionally, consistent results were confirmed even after changing the observation period for avoidable diabetes-related hospitalization to two- and three-year in the sensitivity analysis. Despite these limitations, this study is significant in addressing the short- and long-term associations between avoidable diabetes-related hospitalizations and health expenditures among older patients with T2DM. Furthermore, the IPTW method contributes to obtaining analytical results with a high level of evidence.

## 5 Conclusion

Older patients with T2DM who experienced avoidable diabetes-related hospitalization have an increased risk of incurring higher short- and long-term health expenditures. Therefore, there is a need for continuous health management to prevent avoidable hospitalization, thereby promoting health and ensuring the financial stability of older patients with T2DM within the healthcare insurance system.

## Funding

This work was supported by the National Health Insurance Service Ilsan Hospital [grant no. NHIMC-2023-CR-055].

## Ethical approval

Ethical approval for this study was waived by the Institutional Review Board of the National Health Insurance Service Ilsan Hospital, South Korea (NHIMC 2024–01-025), as it used only secondary data with anonymized and encrypted personal information.

## Source of data

This study used NHIS-Senior data (NHIS-2024-2-027) made by National Health Insurance Service (NHIS). The authors declare no conflict of interest with NHIS.

## CRediT authorship contribution statement

**Woo-Ri Lee:** Writing – review & editing, Writing – original draft, Methodology, Formal analysis, Conceptualization. **Gyeong-Min Lee:** Writing – review & editing, Writing – original draft, Conceptualization. **Noorhee Son:** Writing – original draft, Methodology, Conceptualization. **Kyu-Tae Han:** Writing – original draft, Methodology, Conceptualization. **Sungyoun Chun:** Writing – original draft, Conceptualization. **Yehrhee Son:** Writing – original draft, Conceptualization. **Ki-Bong Yoo:** Writing – review & editing, Writing – original draft, Methodology, Conceptualization.

## Declaration of competing interest

The authors declare that they have no known competing financial interests or personal relationships that could have appeared to influence the work reported in this paper.

## Data Availability

Data for this study are public secondary data, and it can be accessed through the following NHIS website by submitting an application form and paying a fee (https://nhiss.nhis.or.kr).

## Appendix A Supplementary data

Supplementary material Image 1
