# Supplementary material for: Is avoidable diabetes-related hospitalization in older patients with type 2 diabetes mellitus associated with increased health expenditure?: A nationwide retrospective cohort study in South Korea
Source: Prev Med Rep. 2024 Dec 15;49:102946. doi: 10.1016/j.pmedr.2024.102946 (PMC11729008; doi:10.1016/j.pmedr.2024.102946)
Supplement: Supplementary file 1 — Supplementary material [file mmc1.docx]

| **Supplementary Table 1. Differences in covariates’ standardized difference of the inverse probability of treatment weighting model using National Health Insurance Services-Senior cohort 2008–2019 in South Korea** | | | | |
| --- | --- | --- | --- | --- |
| **Variable** | **Standardized difference** | | | |
|  | **Normal model** | **Two years** | **Three years** | **T2DM definition change** |
|  |  |  |  |  |
| **Sex** |  |  |  |  |
| Male |  |  |  |  |
| Female | <0.01 | -0.04 | -0.01 | -0.03 |
| **Age** |  |  |  |  |
| 60s |  |  |  |  |
| 70s | 0.02 | 0.05 | 0.01 | <0.01 |
| ≥80s | 0.02 | 0.04 | 0.03 | <0.01 |
| **Income** |  |  |  |  |
| ≤20 % (lowest) |  |  |  |  |
| 21 %–50 % | <0.01 | -0.01 | <0.01 | <0.01 |
| 51 %–80 % | <0.01 | 0.08 | 0.05 | 0.02 |
| 81 %–100 % (highest) | -0.01 | -0.05 | -0.07 | -0.04 |
| **Region** |  |  |  |  |
| Seoul |  |  |  |  |
| Gyeonggi | -0.06 | -0.03 | 0.02 | -0.07 |
| Metropolitan | -0.02 | -0.05 | -0.04 | 0.01 |
| Other cities | 0.02 | 0.06 | 0.05 | 0.02 |
| **Type of healthcare insurance** |  |  |  |  |
| Medical Aid |  |  |  |  |
| National Health Insurance | 0.01 | 0.01 | -0.03 | -0.01 |
| **Disability** |  |  |  |  |
| Non-disabled |  |  |  |  |
| Disabled | -0.01 | 0.03 | 0.02 | -0.01 |
| **CCI** |  |  |  |  |
| 0 |  |  |  |  |
| 1 | -0.02 | 0.03 | -0.01 | -0.03 |
| 2 | -0.02 | -0.03 | 0.02 | <0.01 |
| ≥3 | 0.05 | 0.07 | 0.04 | 0.05 |
| **DCSI** |  |  |  |  |
| 0 |  |  |  |  |
| 1 | <0.01 | 0.03 | 0.03 | <0.01 |
| 2 | 0.05 | 0.05 | 0.03 | 0.04 |
| ≥3 | 0.04 | -0.01 | -0.01 | 0.03 |
| **Continuity of Care** |  |  |  |  |
| Bad (<1) |  |  |  |  |
| Good (=1) | -0.01 | 0.01 | -0.02 | -0.01 |
| **Death** |  |  |  |  |
| No |  |  |  |  |
| Yes | 0.02 | -0.02 | <0.01 | 0.02 |
| **Diagnosed year of T2DM** |  |  |  |  |
| 2008 |  |  |  |  |
| 2009 | 0.02 | -0.05 | -0.04 | 0.03 |
| 2010 | 0.03 | 0.02 | 0.03 | 0.03 |
| 2011 | 0.02 | -0.04 | -0.01 | 0.01 |
| 2012 | -0.04 | -0.03 | -0.04 | -0.04 |
| 2013 | 0.01 | 0.06 | 0.03 | -0.01 |
| **HR: Hazard risk; CI: confidence interval; CCI: Charlson comorbidity index; DCSI: Diabetes complication severity index; T2DM: Type 2 diabetes mellitus* | | | | |

| **Supplementary Table 2. Generalized estimating equations analysis using a normal distribution on the association between avoidable diabetes-related hospitalization and health expenditure using National Health Insurance Services-Senior cohort 2008–2019 in South Korea** | | | | | | | | |
| --- | --- | --- | --- | --- | --- | --- | --- | --- |
| **Variable** | **Health expenditure (Unit: USD)** | | | | | | | |
|  | **Unweighted model** | | | | **IPTW weighted model** | | | |
|  | **One year** | | **Five years** | | **One year** | | **Five years** | |
|  | **β** | **p-value** | **β** | **p-value** | **β** | **p-value** | **β** | **p-value** |
| **Avoidable diabetes-related hospitalization** |  |  |  |  |  |  |  |  |
| No | Ref |  | Ref |  | Ref |  | Ref |  |
| Yes | 21 | <0.01 | 8262 | <0.01 | 2106 | <0.01 | 8531 | <0.01 |
| **Sex** |  |  |  |  |  |  |  |  |
| Male | Ref |  | Ref |  | Ref |  | Ref |  |
| Female | -115 | 0.06 | 165 | 0.47 | -156 | 0.03 | 75 | 0.78 |
| **Age** |  |  |  |  |  |  |  |  |
| 60s | Ref |  | Ref |  | Ref |  | Ref |  |
| 70s | 418 | <0.01 | 1899 | <0.01 | 574 | <0.01 | 2003 | <0.01 |
| ≥80s | 682 | <0.01 | 2234 | <0.01 | 864 | <0.01 | 3902 | <0.01 |
| **Income** |  |  |  |  |  |  |  |  |
| ≤20 % (lowest) | Ref |  | Ref |  | Ref |  | Ref |  |
| 21 %–50 % | -125 | 0.22 | -76 | 0.84 | -283 | 0.02 | -519 | 0.24 |
| 51 %–80 % | 65 | 0.47 | -20 | 0.95 | 3 | 0.98 | -341 | 0.40 |
| 81 %–100 % (highest) | 126 | 0.16 | 267 | 0.44 | -31 | 0.77 | -275 | 0.49 |
| **Region** |  |  |  |  |  |  |  |  |
| Seoul | Ref |  | Ref |  | Ref |  | Ref |  |
| Gyeonggi | 108 | 0.25 | -589 | 0.10 | 282 | 0.01 | -1126 | <0.01 |
| Metropolitan | 177 | 0.04 | 636 | 0.06 | 310 | <0.01 | 965 | 0.02 |
| Other cities | 164 | 0.04 | 788 | 0.01 | 169 | 0.09 | 792 | 0.03 |
| **Type of healthcare insurance** |  |  |  |  |  |  |  |  |
| Medical Aid | Ref |  | Ref |  | Ref |  | Ref |  |
| National Health Insurance | -619 | <0.01 | -2736 | <0.01 | -586 | <0.01 | -3213 | <0.01 |
| **Disability** |  |  |  |  |  |  |  |  |
| Non-disabled | Ref |  | Ref |  | Ref |  | Ref |  |
| Disabled | 885 | <0.01 | 3196 | <0.01 | 1033 | <0.01 | 3112 | <0.01 |
| **CCI** |  |  |  |  |  |  |  |  |
| 0 | Ref |  | Ref |  | Ref |  | Ref |  |
| 1 | 438 | <0.01 | 2062 | <0.01 | 520 | <0.01 | 2503 | <0.01 |
| 2 | 346 | <0.01 | 1731 | <0.01 | 296 | <0.01 | 1763 | <0.01 |
| ≥3 | 1,328 | <0.01 | 4642 | <0.01 | 1558 | <0.01 | 5401 | <0.01 |
| **DCSI** |  |  |  |  |  |  |  |  |
| 0 | Ref |  | Ref |  | Ref |  | Ref |  |
| 1 | -266 | 0.03 | -1,945 | <0.01 | 138 | 0.31 | -2029 | <0.01 |
| 2 | -378 | 0.04 | -2,211 | <0.01 | -512 | 0.01 | -3114 | <0.01 |
| ≥3 | 324 | 0.14 | -497 | 0.55 | 148 | 0.50 | -2,99 | <0.01 |
| **Continuity of Care** |  |  |  |  |  |  |  |  |
| Bad (<1) | Ref |  | Ref |  | Ref |  | Ref |  |
| Good (=1) | -202 | <0.01 | -998 | <0.01 | -85 | 0.25 | -617 | 0.03 |
| **Death** |  |  |  |  |  |  |  |  |
| No | Ref |  | Ref |  | Ref |  | Ref |  |
| Yes | 624 | <0.01 | 13,871 | <0.01 | -270 | 0.25 | 12,350 | <0.01 |
| **Diagnosed year of T2DM** |  |  |  |  |  |  |  |  |
| 2008 | Ref |  | Ref |  | Ref |  | Ref |  |
| 2009 | 155 | 0.13 | -399 | 0.31 | 65 | 0.59 | -1563 | <0.01 |
| 2010 | 16 | 0.87 | -374 | 0.34 | 93 | 0.43 | -961 | 0.03 |
| 2011 | 161 | 0.11 | 462 | 0.23 | 165 | 0.16 | -159 | 0.72 |
| 2012 | 39 | 0.70 | 315 | 0.41 | -14 | 0.91 | -398 | 0.37 |
| 2013 | -32 | 0.76 | 772 | 0.04 | 352 | <0.01 | 1517 | <0.01 |
| **IPTW: Inverse probability of treatment weighting; Ref: Reference; CCI: Charlson comorbidity index; DCSI: Diabetes complication severity index; T2DM: Type 2 diabetes mellitus*  ***p-value were generated using generalized estimating equations analysis* | | | | | | | | |
